# Women Who Sexually Abuse Children – Results From an Anonymous Online Survey Among a Non-Forensic Female Sample With Sexual Interest in Children

**DOI:** 10.5964/sotrap.14741

**Published:** 2025-08-21

**Authors:** Safiye Tozdan, Peer Briken, Johanna Schröder

**Affiliations:** 1 Institute for Sex Research, Sexual Medicine and Forensic Psychiatry, University Medical Center Hamburg-Eppendorf, Hamburg, Germany; 2 Institute for Clinical Psychology and Psychotherapy, Department of Psychology, Medical School Hamburg, Hamburg, Germany; Centre for Criminology (Kriminologische Zentralstelle – KrimZ), Wiesbaden, Germany

**Keywords:** female pedophilia, female hebephilia, sexual violence against children, female sex offenders, female child sexual offenders

## Abstract

Knowledge is still rare regarding women who commit child sexual abuse (CSA), especially those who have a sexual interest in children. The present study aimed at assessing characteristics of CSA committed by women. We investigated self-report data from 23 German women from the general population who reported a sexual interest in children and previous acts of CSA. Results showed that participants were mostly well-educated with almost no reported childhood maltreatment. At first offence, participants were 25.22 years and children (65.2% female) were 5.30 years on average. 95.5% of participants were known or related to the child and all of them conducted at least one contact sexual offense. 87% stated they conducted the sexual acts for “sexual satisfaction”; 34.8% out of “love”; 30.4% for “physical closeness”; and 34.8% for “emotional closeness”. The results indicate that participants conduct the offence voluntarily and were not coerced by anyone else to engage in CSA. Our results might indicate that women who commit CSA and have a sexual interest in children may constitute a specific subtype that differs in several ways from forensic samples of women committing CSA examined in the past.

## Abstract (plain language summary)

**Background:**

Child sexual abuse (CSA) is often viewed as a crime perpetrated solely by men, but research indicates that women also commit CSA offenses, significantly affecting children’s health and wellbeing. Although research on women who sexually offend against children has increased over the past thirty years, it remains limited compared to studies on male perpetrators. Notably, to the authors’ knowledge, there have been no investigations of women who report sexual interest in children and CSA offenses.

**Why was this study done?:**

The present study was done to better understand the nature of CSA perpetrated by women with sexual interest in children. The findings of this study are valuable as they inform the development of more effective interventions and prevention strategies for female individuals at risk of offending in the context of CSA.

**What did the researchers do and find?:**

We conducted an anonymous online survey with 23 German speaking women from the general population who reported having a sexual interest in children and reported previous sexual acts with children under the age of 14 years. The findings revealed that these women were mostly well-educated and rarely reported own adverse childhood experiences, which contradicts previous findings on women who have been convicted of CSA. On average, the women in our study were 25 years old at the time of their first offense, while the children affected were on average 5 years old, with 65% being female. Almost all of the participants knew the child or were related to them, and all reported having committed at least one sexual offense involving physical contact. The reasons they gave to explain their actions included seeking sexual satisfaction, feelings of love, desires for physical closeness, and emotional closeness. Our results further suggest that the participants acted voluntarily and were not pressured by anyone else to commit the crime.

**What do these findings mean?:**

Despite the limitations posed by the small sample size, the present results can contribute to a better understanding of women who commit sexual offenses against children. Our findings suggest that the current portrayal of women who engage in CSA may not fully capture the entire population of women who sexually offend against children, as it might overlook those who likely operate under the radar of criminal prosecution.

## Highlights

- Our results contradict previous findings that women who have been convicted of CSA tend to have a low level of education.
- Our results are not in line with previous findings demonstrating that women who have been convicted of CSA tend to report their own childhood maltreatment.
- Our results confirm that sexual interest in children as a motive for sexually abusive acts against children seems to be also relevant in women.

Child sexual abuse (CSA) occurs in a variety of cultures among different socioeconomic backgrounds and comprises distinguished types of sexual offences against children including physical and mental violence (Murray et al., 2014). According to two international meta-analyses, up to 20% of girls and 8% of boys are affected by CSA (Pereda et al., 2009; Stoltenborgh et al., 2011). For a long time, CSA has been conceptualized as a male-perpetrated offence (Denov, 2003). However, it has been established nowadays that females also engage in sexually assaultive behavior with severe impact on the affected individual (Cortoni et al., 2017). Research on CSA committed by women has grown over the last thirty years (e.g., Augarde & Rydon-Grange, 2022) and focused on samples from incarcerated women who committed sexual crimes against children. However, the state of research on female-perpetrated CSA is still insufficient compared to research on male perpetrated CSA. Especially, to our knowledge, no study so far investigated a non-forensic sample of women (i.e., women from the general population) who self-report they sexually abused children and have a sexual interest in children. The present study therefore adds to this research gap by providing data from an anonymous online study among women with sexual interest in children who committed CSA. Ultimately, such results could be helpful in the planning and implementation of CSA interventions and prevention programs for individuals who are at risk to sexually offend against children.

## Prevalence of Female-Perpetrated CSA

It can be assumed that the actual proportion of female-perpetrated CSA is barely represented by official reports (i.e., from police or court offices) (Tozdan et al., 2019). For instance, official crime statistics in Canada showed that 3.7% of sexual offences against children in 2017 (total *N* of accused persons for sexual violations specific to children = 4,703) were committed by women (Savage, 2019). The Australian Bureau of Statistics reported that in 2005, 1.7% of sexual offences against children under the age of 15 years (total estimated n of people Australian people who had experienced sexual abuse before the age of 15 years = 1,294,000) were committed by women (Richards, 2011). German crime statistics showed that 712 adults have been convicted for CSA in 2020 of which 1.5% have been female (Statistisches Bundesamt, 2021). Studies among individuals affected by CSA usually reveal a higher proportion of female-perpetrated abuse. For example, based on reports to the US child protection services, McLeod and Craft (2015) reported a proportion of 20% for female-perpetrated CSA (total *N* of CSA cases = 66,765). Analyses of call records from a free hotline for children in need in the UK in 2008 and 2009 revealed a proportion of 17% for female-perpetrated CSA (total *N* of CSA cases = 12,268) (NSPCC, 2009). The meaningfulness of these results is however limited, as the data is not representative. Representative data rather reveals lower proportion rates. For instance, Bourke et al. (2014) examined data from a nationally representative survey investigating sexual violence among Irish adults and reported a proportion of 6% for CSA committed by women of the total amount of CSA cases (total *N* of CSA cases = 3,120) (Bourke et al., 2014). Tozdan et al. (2021) examined representative self-report data collected in Germany and found a proportion of 9% for self-reported female-perpetrated sexual abuse against male minors (total *N* of sexually abused male minors = 87). It has to be mentioned that the differences in all prevalence rates reported here are partly caused by different methods, populations investigated, and definition of CSA. Nevertheless, even the rates found in representative data asking individuals affected by CSA suggest an underestimation of the problem in data from official crime reports, too. Thus, female-perpetrated CSA cannot be seen as a rare phenomenon, but rather as an under-reported and under-detected crime (Tozdan et al., 2019).

## Characteristics of Women Committing CSA

Although women who sexually abuse children are considered as rather heterogeneous population with different features (Gannon & Rose, 2008; Miccio-Fonseca, 2000; Sandler & Freeman, 2007; Vandiver & Kercher, 2004), some common characteristics of these women and their offences were found. The average age of women committing CSA seems to range from 20 to 36 years (Gannon & Rose, 2008; Wijkman et al., 2011). Research mainly demonstrated that they have a rather low socioeconomic status (e.g., Gannon & Rose, 2008; Nathan & Ward, 2001; Lewis & Stanley, 2000) with little vocational qualifications (e.g., Nathan & Ward, 2001; Tardif et al., 2005). Furthermore, they often experienced mental, sexual, and/or physical abuse themselves during childhood (e.g., Lewis & Stanley, 2000; Nathan & Ward, 2001) and suffer from various mental health problems including substance abuse and depression (e.g., Berner et al., 2022). In addition, they are frequently involved in abusive relationships during adulthood (e.g., Lewis & Stanley, 2000; Matthews, 1993).

The abused children’s ages range from infants to adolescents (Briggs & Hawkins, 1995; Ogilvie & Daniluk, 1995). Among many studies, CSA committed by women was shown to be more prevalent among affected males than affected females (e.g., Cortoni et al., 2017; Dube et al., 2005; NSPCC, 2009; Stadler et al., 2011). However, other studies revealed that women prefer female children (e.g., McLeod, 2015; Tardif et al., 2005) and others suggest that women do not have a preferred gender of the abused children (e.g., Colson et al., 2013; Johansson-Love & Fremouw, 2006, 2009; Lewis & Stanley, 2000). Other research results indicate that the children’s gender may be determined by whether the woman is accompanied by a male co-offender (Muskens et al., 2011; ten Bensel et al., 2019; Vandiver, 2006). This means, women who act alone seem to be more likely to abuse male children whereas women who act with a male partner seem to be more likely to offend against female children. Moreover, women who abuse children often are related or at least known to the child. They are the children’s caregivers, i.e., mothers, other relatives, or babysitters (Lewis & Stanley, 2000; Vandiver & Walker, 2002).

With regard to sexual acts that women commit against children, it was found that women – similar to men – show a wide range of different acts. These include, for example, vaginal and anal penetration, active and passive oral sex, touching or caressing genitals, exposure of genitals, exploitation for prostitution, production of abuse images, and sexual harassment (e.g., Gewitz-Meydan et al., 2023; Peter, 2009). Furthermore, studies demonstrate that some of the women apparently act out of sadistic motives and use a high degree of physical violence (e.g., Gebhardt et al., 2022; Gewitz-Meydan et al., 2023).

Although sexual interest in children is considered to be a meaningful risk factor for sexual offence recidivism in male samples (Mann et al., 2010), sexually offending against children cannot be considered as a reliable indicator of sexual interest in children (Wollert & Cramer, 2011). It needs to be mentioned that findings on male sample cannot simply be transferred to women. However, being sexually aroused by children seems to be a motive for women to commit CSA, too (Brown & Kloess, 2020). For example, Chow and Choy (2002) reported on a 23-year-old woman who sexually abused the 4-year-old daughter of a friend. While bathing the little girl, the woman became aroused by touching her. After the bath, she took the girl into the bedroom, spread her legs and licked the girl’s vaginal area for a few minutes. This was sexually gratifying to her (Chow & Choy, 2002). However, pedophilic interest in females has been neglected in research for a long time compared to pedophilic interest in males giving the impression that pedophilic women rarely exist. Recent research finally starts to focus on examining women who have a sexual interest in children (e.g., Lievesley & Lapworth, 2022). Other motives of women for sexual offences against children that have been revealed by research include the feeling of control and power, the need for intimacy and closeness, and the desire for acceptance and attention (Brown & Kloess, 2020).

The results presented above are based on samples of women convicted of CSA. To our knowledge, no study so far solely aimed at investigating CSA committed by women who report to have a sexual interest in children.

## The Present Study

Knowledge is still rare regarding offence characteristics of women for committing CSA. We therefore focused on assessing characteristics of CSA committed by women by investigating self-report data via a German survey gathered online among a sample of women from the general population who self-identified as having a sexual interest in children and reported previous sexual contacts with children under the age of 14 years. The present sample is a subsample of the participants reported by Tozdan et al. (2022), Erkan et al. (2024), and Tozdan et al. (2025). The vast majority of results reported in the present study are however published for the first time.

## Materials and Method

### Procedure

The data was collected via a German online survey from July to December 2020 among women. One study link was generated with Qualtrics (www.qualtrics.com); a second one was generated with LimeSurvey (www.limesurvey.org) allowing participants to conduct the survey via browsers guaranteeing more anonymity, such as the “Tor Browser”. The study links were mainly spread on websites directed towards individuals with sexual interest in children and/or that provide information on pedophilia.^1^1www.gsa-forum.de, www.kinder-im-herzen.net, www.schicksal-und-herausforderung.de, www.paedoseite.home.blog, www.krumme13.org, www.virped.org. Most of these websites are run by individuals who clearly speak out against CSA as they do not want to harm children. We also spread the links via the homepage of the research project, the Instagram account of the University Medical Center Hamburg-Eppendorf, emails to the German psychotherapist’s chambers, and several general websites.^2^2www.gute-frage.net, www.paradisi.de, www.psychologie.gofeminin.de, www.urbia.de. In addition, staff members of the outpatient treatment center of the Institute for Sex Research, Sexual Medicine, and Forensic Psychiatry at the University Medical Center Hamburg-Eppendorf and the counselling center “Wendepunkt e.V.” for minors and young adults with sexually conspicuous behavior in Hamburg were informed about the study.

The title of the study was “Unknown female sexuality? Sexual interest in children and/or adolescents”. The original aim was to investigate women who had a sexual interest in children. For this reason, the survey was addressed at women with a sexual interest in children and not at female perpetrators of CSA. However, we took the opportunity to also ask participants about previous sexual acts with children. At the beginning of the survey, participants were informed that the anonymous survey is directed to females who are at least 18 years of age and are sexually interested in children under the age of 14. Informed consent was obtained from all participants via mouse click. The ethics committee of the University Medical-Centre Hamburg (reference number: LPEK-0110) approved the study.

### Participants

Inclusion criteria for the study (Tozdan et al., 2022) were (1) being born as a female or identifying with the female gender, (2) being at least 18 years old, and (3) having a self-identified sexual interest in children under the age of 14 years. Exclusion criterion was identifying solely with the male gender. During the survey, participants were asked if they have had sexual contact with children less than 14 years. For the purpose of the present study, we only analyzed participants who reported previous sexual contact with children under the age of 14. Thus, the present sample consists of 23 participants. Table 1 shows sample characteristics.

**Table 1:** Sample Characteristics for the Total Sample as Well as for Female Born and Male or Other Born Participants

Variable	Total sample (*N* = 23)		Female born (*n* = 19)		Male/other born (*n* = 4)	
	*M*	*SD*	*Mdn*		*Mdn*	
**Age at data collection**	35.61	9.24	36		27	
	***n***	**%**	***n***	**%**	***n***	**%**
Education level						
Low	8	34.8	7	36.8	1	25.0
Moderate	4	17.4	3	15.8	1	25.0
High	11	47.8	9	47.4	2	50.0
Steady relationship						
No	12	52.2	9	47.4	3	75.0
Yes	11	47.8	10	52.6	1	25.0
Ever been diagnosed with a mental disorder						
No	18	78.3	15	78.9	3	75.0
Yes	5	21.7	4	21.1	1	25.0
Indication of ICD-11 Pedophilic Disorder						
No	5	21.7	3	15.8	2	50.0
Yes	18	78.3	16	84.2	2	50.0
	*M*	*SD*	*M*	*SD*	*M*	*SD*
**ACE score**	2.56	1.65	2	0-5	3.5	3-4

A total of 19 participants reported that they were born as a female and identify with the female gender. A total of three participants reported being born as a male. Of those, two participants indicated that they identify with the female gender and one indicated identifying with both male and female gender. One participant reported another gender at birth and indicated identifying with both male and female gender.^3^3It needs to be mentioned at this point that research indicates that biological men and biological women should not be treated equally in terms of sexual (offending) behavior (e.g., Lawrence et al., 2005; Raines et al., 2021). This is why we chose to also report the results for both subsamples (female born and male/other born) separately. Our results demonstrated that the inclusion of the four male/other born participants did not change the results substantially and thus, the discussion and conclusion of the present study would have been the same. This is why we refuse to exclude participants who identified with the female gender and felt addressed by our study. Furthermore, for the total sample, participants’ ages at data collection ranged from 24 to 64 years (female born = 26-64; male/other born = 24-50). Diagnoses reported by participants included depression (*n* = 4), cannabis addiction (*n* = 1), combined personality disorder (*n* = 1), impulse control disorder (*n* = 1), anxiety disorder (*n* = 1), posttraumatic stress disorder (*n* = 1), and Munchhausen by proxy syndrome incl. Munchhausen by adult proxy syndrome (*n* = 1)^4^4This was the original wording of the participant which we prefer to choose for the report. However, the actual diagnosis would be “factitious disorder imposed on another” (World Health Organization, 2020)..

### Measures

Sample characteristics (age at data collection, education level, relationship status, diagnosis of mental disorder) were assessed. We further assessed childhood maltreatment using the German version of the “Adverse Childhood Experiences Questionnaire” (ACE-D; Schäfer et al., 2014). This 10-item instrument captured participants’ adverse childhood experiences before the age of 18 (emotional, physical, and sexual abuse; emotional and physical neglect; household dysfunction). Higher scores indicate more severe adverse experiences during childhood. The questionnaire has a total score of 10. For the purpose of the present study, we included nine self-generated questions in order to ascertain offence characteristics (sexual contact with children under 14, children’s and participant’s age at offence, child’s gender, relationship to child, sexual acts committed, motivation for sexual acts, evaluation of sexual acts, open question on further comments on sexual acts with children) and one question to assess an indication of pedophilic disorder diagnosis. For replicable results, we described these questions in detail in the Supplementary Materials of this article (see Tozdan et al., 2025S).

### Data Analyses and Presentation

Data analyses were performed using IBM SPSS Statistics, version 22 (International Business Machines Corp., 2013). As four participants were not born female who may have an impact on the results, we presented the results for the total sample and for the two subsamples, “female born” and “male/other born”. Comparison analyses between the two subsamples were considered informative, but were not conducted as the “male/other born” subsample was too small. Since we recognized that adding the male/other subsample to the total sample did not have a substantial impact on the total results, we did not discuss the results for both subsample but only for the total sample. Quantitative data was assessed descriptively only (mean value, standard deviation, and median for interval scaled variables; frequency and percentages for categorical and nominal variables). Qualitative data from free text fields was all reported attempting to sort them into groups.

## Results

Results are shown in Table 2. Five participants – who were all female born – did not fill in the ACE. The ACE score for the total sample ranged from 0 to 5 (female born: 0-5, male/other born: 3-4). Participant’s age at offence ranged from 18 to 40 years (female born: 18-40 years, male/other born: 18-27 years). Child’s age at offence ranged from 1 to 13 years (female born: 1-13 years, male/other born: 1-12 years).

**Table 2:** Descriptive Statistics for Results Concerning the Sexual Abuse of Children for the Total Sample as Well as for Female Born and Male or Other Born Participants

Variable	Total sample (*N* = 23)		Female born (*n* = 19)		Male/other born (*n* = 4)	
	*M*	*SD*	*Mdn*		*Mdn*	
**Participant’s age at offence**	25.22	5.92	25.0		21.5	
**Child’s age at offence**	5.30	4.03	3.5		7.5	
	*n*	%	*n*	%	*n*	%
Child’s gender						
Female	15	65.2	12	66.7	3	75.0
Male	7	30.4	6	33.3	1	25.0
N/A	1	4.3	1	5.3	–	
Relationship to child						
Relative	10	43.5	8	42.1	2	50.0
Known person	12	52.0	10	52.6	2	50.0
Unknown person	1	4.3	1	5.3	–	
Sexual acts (multiple answers allowed)						
I exposed my vagina to this person to arouse myself sexually.	14	60.9	13	68.4	1	25.0
I asked the person to touch my vagina or to sexually arouse me otherwise with the hand or the mouth.	13	56.5	12	63.2	1	25.0
I touched the person’s genitals (vagina or penis), the chest or the anus (buttocks) to arouse myself sexually.	16	69.6	13	68.4	3	75.0
I touched the person in other areas of the body to arouse myself sexually.	10	43.5	8	42.1	2	50.0
I masturbated in front of this person with the knowledge that he or she was aware of it.	15	65.2	14	73.7	1	25.0
I inserted my finger, my tongue or an object into the person’s vagina or anus (buttocks).	14	60.9	10	52.6	4	100
I created pornographic material (“porn” in the form of videos, photos or audios) of the person.	3	13.0	3	15.8	–	
I asked the person to watch pornographic material (“porn”) with me.	6	26.1	4	21.1	2	50.0
I asked the person to engage in sexual acts with someone else.	2	8.7	2	10.5	–	
I watched someone else engage in sexual acts with the person.	8	34.8	7	36.8	1	25.0
I was pushy towards the person (e.g. by kissing or touching against the person’s will).	11	47.8	10	52.6	1	25.0
I threatened the person or blackmailed the person so that he or she would engage in sexual acts with me.	2	8.7	2	10.5	–	
I gave the person alcohol or drugs so that he or she would engage in sexual acts with me.	2	8.7	2	10.5	–	
I was violent towards the person (e.g., by hitting, tying them up, ...) so that he or she would engage in sexual acts with me.	4	17.4	3	15.8	1	25.0
I engaged in other sexual acts with the person.	8	34.8	4	21.1	1	25.0
Sexual acts, at least one…(multiple categories possible)						
Contact sexual offenses	23	100	19	100	4	100
Non-contact sexual offenses	17	73.9	15	78.9	2	50.0
Non-sexual abuse supporting acts	5	21.7	4	21.1	1	25.0
Motivation for sexual acts (multiple answers allowed)						
Out of love	8	34.8	6	31.6	2	50.0
To satisfy myself sexually	20	87.0	17	89.5	3	75.0
To be physically closer to the person	7	30.4	4	21.1	3	75.0
To be closer to the person emotionally (in terms of feelings)	8	34.8	6	31.6	2	50.0
To show the person that I had power over him or her	4	17.4	3	15.8	1	25.0
Other	7	30.4	6	31.6	1	25.0
Evaluation of sexual acts						
The sexual acts were carried out with mutual consent, i.e., the other person and I both agreed to them.	10	43.5	7	36.8	3	75.0
The sexual acts were not carried out with mutual consent because …	13	56.5	12	66.7	1	25.0
... the other person didn’t consent.	11	47.8	10	52.6	1	25.0
... I didn’t consent.	–		–		–	
... neither the other person nor I consented.	2	8.7	2	10.5	–	
**Further comments**	12	52.2	11	57.9	1	25.0

### Motivation for Sexual Acts

Three participants indicated their “other” motives for the sexual contact were related to their partners:

“To satisfy my girlfriend.” (This participant was male/other born.)

“To satisfy my boyfriend.”

“Because it excited me to fulfil my partner's wish.”

Two participants specified some sort of intrinsic coercive force:

“It was like I didn't control myself in that moment and like I'm being told inside I have to do it to find out if I'm into something like this.”

“Inner compulsion.”

One participant mentioned the sexual satisfaction of the child:

“To satisfy the person.”

And one participant reported different motives:

“Desire for the dissolving of boundaries and merging and pure, innocent happiness and love [.] Longing for regression into the still happy and unencumbered childhood.”

### Further Comments on Sexual Acts With Children

Three participants referred to their (sexual) arousal:

“Arousing.”

“Had orgasms.”

“I was excited.”

Three participants made statements about the child’s consensus:

“The girl was the daughter of my boyfriend at the time. The sex was sometimes consensual and sometimes non-consensual.”

“The boy had asked me – I agreed and then it started.” (This participant was male/other born.)

“She wanted to do it, she provoked me at first, I let myself be carried away by this impulse, today we are inseparable.”

Two participants described specific sexual acts:

“I once briefly caressed over the pants in the genital area. Another time when changing I briefly spread the labia to look at the vagina.”

“Mainly joint and mutual masturbation. Closeness. Cuddling with more.”

Two participants indicated negative emotions; an inner conflict including shame:

“Much of the time power and energy represents the inner struggle between on the one hand moral responsibility and bad conscience and on the other hand the urge of excitement and the desire to break morality and taboo[.] shame guilt overcoming enjoyment.”

“I am ashamed.”

One participant highlighted the hiding of the sexual contact:

“Would have been nice if you didn't have to hide, would have been great for both of us.”

Finally, one participant commented on children’s sexuality in general:

“Without false social taboos and moral concepts, sexuality is always something happy and natural, even for small children, and all children enjoy intensive physical attention and love and are even crazy about it if they are introduced to them in a child-friendly, playful and fearless manner and if they are rewarded[.] and when you build a good, secure and very special appreciative relationship[.] respond to their great need, to their unfulfilled wishes and longings[.] A lot of time tenderness intimacy empathy emotional affirmation appreciation love happiness.”

## Discussion

### Sample Characteristics

The present study investigated 23 women who have a self-identified sexual interest in children and reported sexual abuse of children under 14 via an anonymous German online survey. About half of the present participants (47.8%) reported a high education level which is not in line with research showing that women who commit CSA mainly demonstrate a rather low educational status (e.g., Gannon & Rose, 2008; Lewis & Stanley, 2000; Nathan & Ward, 2001). The reason for this may probably be that most research on women who sexually offend against children is based on samples of women who were incarcerated for their crimes. Our study was conducted as an online survey via a non-forensic sample and it is unknown whether the participants had been convicted for their sexual acts with children because the survey did not ask that question.

Women who commit CSA appear to be frequently involved in abusive relationships during adulthood (e.g., Lewis & Stanley, 2000; Matthews, 1993). Half of the present sample (47.8%) mentioned being in a steady relationship. However, it is not possible to conclude whether these relationships are abusive because this was not asked.

Only about a fifth of participants (21.7%) reported being diagnosed with a psychiatric disorder by a mental health professional. This is not in line with research demonstrating that women who sexually offend against children usually suffer from various mental health problems (e.g., Berner et al., 2022). However, most of the women (82.6%) have not sought help from health professionals and thus, probably have never been in the situation of being evaluated by a physician or psychologist. Participants further reported a rather low mean level of childhood maltreatment. Previous results on women sexually offending against children often have shown that they experienced mental, sexual and/or physical abuse themselves during childhood (e.g., Lewis & Stanley, 2000; Nathan & Ward, 2001). Again, this is probably caused by the fact that our sample differs from most previous samples investigated, as the present sample only includes those offending women who have a sexual interest in children.

### Indication of ICD-11 Pedophilic Disorder

At least, the majority of participants (78.3%) had an indication of the ICD-11 Pedophilic Disorder diagnosis, reporting that they experience an intense and persistent sexual interest in prepubescent children. As the study was directed to women with a sexual interest in children, it seems logical that most women tend to meet the diagnostic criteria for pedophilia. In addition, the five participants who did not show such indication might only have a sexual interest in pubescent children. A post hoc descriptive analysis confirmed this assumption. Sexual interest in children has long been considered to be a risk factor for sexual offence recidivism in male samples (Mann et al., 2010). Whether a sexual interest in children among women puts them at risk to sexually offend or offend again after a legal sanction is unknown so far. As said before, findings from male samples cannot be directly generalized to women. Nevertheless, it might be that the present participants’ sexual interest in children put them at risk to sexually offend against children. Recent research has also demonstrated that only an exclusive sexual interest in children seems to be related to sexual recidivism while a non-exclusive sexual interest in children is not (Eher et al., 2015). This might explain why, in Erkan et al. (2024), we found that the present sample of women who reported sexual contact to children did not differ from those who did not report such crimes regarding the indication of ICD-11 Pedophilic Disorder (Erkan et al., 2024). Just as with men, there may be women who have a sexual interest in children, which is likely to result in CSA, and there may be women with a sexual interest in children who choose not to offend against children.

### Sexual Abuse of Children

#### Offence Characteristics

At the time of the offence, participants were 25 years on average which is in line with previous research suggesting that the average age of women committing CSA range from 20 to 36 years (Gannon & Rose, 2008; Wijkman et al., 2011). The abused children were 5 years on average at the time of the offence in the present study. Although the age of children abused by women can range from infants to adolescents (Briggs & Hawkins, 1995; Ogilvie & Daniluk, 1995), 5 years appears to be rather young compared to other studies (e.g., McLeod, 2015; Vandiver & Kercher, 2004). This result may be explained by the fact that we asked participants to report on the youngest child they have had sexual contact with. In contrast, this result may suggest that women who conduct CSA and have a sexual interest in children rather abuse younger children compared to the overall population of women who sexually abuse children. More than half of the present participants (65%) stated that the child’s gender was female which is in line with some previous studies showing that sexually abusing women prefer to abuse female children (e.g., McLeod, 2015; Tardif et al., 2005). At the same time, there is evidence that co-offending women have more female victims while solo offending women have more male victims (Muskens et al., 2011; ten Bensel et al., 2019; Vandiver, 2006). Our results may therefore indicate that a significant number of participants acted in collaboration with a male co-offender. However, for the vast majority of participants, there is no evidence in the data to support this conclusion. Our results are consistent with previous research indicating that women who abuse children often are the children’s caregivers, i.e., mothers, other relatives, or babysitters (Gewitz-Meydan et al., 2023; Peter, 2009). Only one participant in the present study said that the child was unknown to her, the rest either was related (43.5%) or known to the child (52.0%). The wide range of different abusive sexual acts that were pointed out by the present sample also fit into current literature (e.g., Bickart et al., 2019). Most important, all participants in the present sample (100%) conducted contact sexual offenses against the child (e.g., asked the child to touch her genitals or touched the child’s genitals) and the majority (73.9%) also conducted no-contact sexual offenses (e.g., masturbated in front of the child or exposed her genitals to the child). Three participants (13.0%) reported on creating pornographic material of the child which confirms research suggesting that women play a role in production and distribution of child abuse material (Bickart et al., 2019).

#### Motivation for Sexual Acts With Children

Since our study was directed to women who have a sexual interest in children, it is not surprising that most of the participants (87.0%) claimed that they “engage in sexual contact with the child to satisfy themselves sexually” (in a multiple choice question). This is consistent with findings demonstrating that women sexually abuse children because they feel sexually aroused by them (Brown & Kloess, 2020; Chow & Choy, 2002). About one third (34.8%) specified that they “engage in sexual acts with the child out of love”. Mostly the same participants also chose the categories on physically closeness, emotional closeness, and sexual satisfaction. Research on men with sexual interest in children indicates that some not only are sexually attracted to children but also have romantic feelings for them (Seto, 2012). Our results suggest that this may also be the case for women who have a sexual interest in children. Seven participants (30.4%) wanted to be physically closer to the child while eight participants (34.8) stated that they wanted to be emotionally closer to the child. The latter result confirms previous findings on women’s need for intimacy and closeness as motive for CSA (Brown & Kloess, 2020). Finally, four participants (17.4%) reported that they sexually abused the child because they wanted to show the child that they had power over him/her. This is in line with previous results showing that feelings of control and power are the reason why some women commit CSA (Brown & Kloess, 2020). Seven participants (30.4%) described further motives. Three of them claimed that they conducted the acts for their partner. We did not find evidence in the data that these three participants have been coerced by their partners to offend against the child. This indicates that women who are co-offenders in CSA are not necessarily coerced by their partners to engage in sexual acts with children and suggests that these three participants deliberately abused a child together with their partner. Furthermore, two participants referred to an intrinsic coercive force that had driven them into the abuse. One of these participants also stated that she conducted the CSA to satisfy herself sexually and both reported symptoms indicating a Pedophilic Disorder according to the ICD-11.

One participant referred to the desire for dissolving boundaries and the “longing for regression into the still happy and unencumbered childhood” as motives for the sexual abuse. This specific answer might represent an emotional congruence with children that has been shown to be related to pedophilic interest among men (McPhail et al., 2018) and which is associated inter alia with an exaggerated cognitive and emotional affiliation with childhood as well as positive views of children and childhood (McPhail et al., 2013). Although results from studies on men should not be broadly applied to women, this may indicate that a construct like emotional congruence with children also exists in women with sexual interest in children.

#### Evaluation of Sexual Acts With Children

In sum, less than half of participants (43.5%) reported that the CSA was carried out with “mutual consent” meaning that both they themselves and the child agreed to the sexual abuse. In turn, over half of them (56.5%) stated the sexually abusive acts were not carried out with “mutual consent”, mostly because the child did not consent. The only two participants who stated that they themselves did not agree were those who reported an intrinsic coercive force but no external force in the sense of another person that coerced them. Since research suggests that about one third of female perpetrators of CSA commit their sexual offenses in company of a male partner (Williams & Bierie, 2015), we included the question on evaluation in order to detect co-offending women who may have been coerced by their partners to engage in CSA. At least, no participant explicitly claimed that they were forced to conduct the sexually abusive acts by another person. However, within a sample of women who sexually offend against children due to their sexual interest in children, it may be reasonable that they did not have to be forced by anyone. As already mentioned by Williams and Bierie (2015), such results are relevant since they suggest that women who sexually offend against children are not just opportunistic abusers, but rather intentional offenders who display what Gannon and colleagues (2014) characterize as an *explicit-approach offense style* aimed at achieving sexual satisfaction, arousal, or other positive outcomes.

#### Further Comments on Sexual Acts With Children

Analyses of the twelve further comments on CSA revealed that three made statements about their arousal. Three participants referred to the child’s consent. One of them explained that the child was her boyfriend’s daughter and that “the sex was sometimes consensual and sometimes non-consensual”. The same participant stated that she wanted to satisfy his boyfriend by engaging in the sexually abusive acts with the child. However, this participant did not claim that she did not consent to the acts on the question on evaluation of the sexual acts, but stated that the child did not consent. The comments of the other two participants sound as if they shift their responsibility to the child: “The boy had asked me..:”; “…she provoked me…”. This reminds of cognitive distortions also found in men who commit CSA that ascribed sexual desire to the child. The child is also considered as initiator of sexual actions and the adult sees himself as the victim of the child (Eberhaut et al., 2020). Cognitive distortions are false assumptions that serve people who commit CSA to justify the abuse to themselves. In addition, previous research has also demonstrated that women who commit CSA show similar cognitions to men who commit CSA, e.g., viewing children as sexual objects and believing that children are capable of enjoying and desiring sex (Beech et al., 2009; Brown & Kloess, 2020). Two participants described specific sexually abusive acts. One of them reported that she sexually abused the child when she changed its diapers indicating that she took advantage of a daily body care situation. This can in fact assumed to be the way how women can easily cover their sexual assaults, namely through daily care situations where they are alone with the child. Because mothers typically provide daily care for children, society allows for a wider range of natural contacts with them (Tsopelas et al., 2012) which can easily be abused by women, for example, during hygiene care where they need to touch the child’s body. The second comment (“…Cuddling with more.”) sounds as if the participant minimized the sexual offence by referring to an alleged closeness indicating that she misrepresented the abuse as a romantic situation with a partner. Again, such cognitive distortions that minimize CSA have also been found in men who sexually abused children (Eberhaut et al., 2020). Two participants referred to feelings of shame and guilt suggesting that they are aware that their actions were wrong. However, for one of the participants such negative feelings do not appear to be stronger than the feelings of “…excitement and the desire to break morality and taboo…”. Finally, one participant wrote that it would have been nice for her and the child if they did not have to hide and one participant commented on children’s sexuality in general describing that sexuality is always something happy and natural for children and that they enjoy physical attention that need to be introduced to children in a child-friendly and playful way. Both comments sound as if the participants held the view that sexual contacts between children and adults should be legalized. This is another cognitive bias that was found in men who sexually abused children (Eberhaut et al., 2020). Moreover, the statements also confirm previous results showing that women who committed CSA have low empathetic concern for their victims and also view the nature of harm caused by their abusive actions as low (Brown & Kloess, 2020).

### Limitations

The major limitation of the present study is that the sample is very small and not representative. Furthermore, the present data was collected online. On the one hand, this implies a high degree of anonymity, fostering participants’ readiness to respond truthfully. On the other hand, there is a certain degree of uncertainty about participants, which limits the validity of the present results. For example, it could be that men pretended to be women, or that participants generally did not answer honestly because they may not have taken the survey seriously. Although we carefully reviewed each data set to ensure that participants' response patterns were understandable and consistent, such factors cannot be ruled out. Moreover, one participant appears to be not German-speaking. Free text fields showed that this participant answered in Portuguese. However, the response pattern of this participant was fully comprehensible and consistent. Therefore, we assume that this participant either understood the German questions but preferred to answer open questions in Portuguese or used a website translation program to participate in our study. In terms of the latter option, we cannot guarantee whether the Portuguese version of our study was translated correctly in all terms. Finally, four participants did not fit the traditional definition of women as being born female. Research has demonstrated that biological men and biological women differ regarding sexual arousal and behavior. For instance, cisgender male pattern of sexual arousal have been found to be comparable with the pattern found in transgender women (Lawrence et al., 2005), while transgender men’s patterns of sexual arousal correspond to those of cisgender women (Raines et al., 2021). This is a strong argument for not mixing male-born individuals who identify as women with cisgender females in terms of sexual offending behavior. This is why we chose to also report the results for both subsamples separately (see Table 1 and Table 2). Additionally, our results clearly showed that the inclusion of the four male/other born participants did not change the results substantially and thus, the discussion and conclusion of the present study would have been the same even if we excluded these participants. Therefore, we decided not to exclude participants who identified with the female gender and felt addressed by our study.

### Conclusion

To our knowledge, this is the first study so far that investigated a non-forensic sample of women who reported they have sexually abused a child. Although the results are limited by the small sample, they might help to better understand women who sexually offend against children. Our results suggest that the current picture of women who conduct CSA may not represent the total population of women who sexually abuse children, as it overlooks those who offend due to sexual interest in children and likely operate outside the reach of the legal system. In general, women who commit CSA need to be taken more seriously, not only by the public in general, but also by professionals in the health care and judicial system as there seem to be women who are at risk to sexually abuse children. The current results might be incorporated into the planning and implementation of CSA interventions and prevention programs. In general, prevention programs need to actively reach out for women with sexual interest in children who may be at risk to sexually offend against children.

## Supplementary Materials

The Supplementary Materials contain a detailed description of the assessed variables for replicable results (see Tozdan et al., 2025S).



TozdanS.
BrikenP.
SchröderJ.
 (2025S). Supplementary materials to "Women who sexually abuse children – Results from an anonymous online survey among a non-forensic female sample with sexual interest in children"
[Additional information]. PsychOpen. 10.23668/psycharchives.19392
PMC1291460141716193

## Data Availability

The data that support the findings of this study are available from the corresponding author, ST, upon reasonable request.

## Author Note

The present sample is a subsample of the participants reported firstly in Tozdan et al. (2022) on women who have a sexual interest in children. While collecting data, we also asked the women about previous sexual acts with children under the age of 14 years. The corresponding data (e.g., child’s age and gender, sexual acts, motives for the abuse etc.) are only published within the present publication. Thus, the vast majority of results reported in the present study are published for the first time. The survey we conducted was very extensive. Previous articles on this sample had a different focus and since most journals have character or page limits, the other articles would have been too extensive if the current variables had been also taken into account.
